# To bind or not to bind: The pelvic2bind cross-sectional survey study

**DOI:** 10.1097/MD.0000000000043724

**Published:** 2025-08-15

**Authors:** Roman Klein, Nikolai Ramadanov, Ali Darwich, Joakim Jørgensen, Arnold J. Suda, Tim Pohlemann, Christoph Wölfl

**Affiliations:** a Department of Orthopaedics, Trauma Surgery and Sports Traumatology, Marienhausklinikum Hetzelstift, Neustadt, Germany; b Center of Orthopaedics and Traumatology, Brandenburg Medical School, University Hospital Brandenburg/Havel, Brandenburg an der Havel, Germany; c Faculty of Health Science Brandenburg, Brandenburg Medical School Theodor Fontane, Brandenburg/Havel, Germany; d Department of Orthopedics and Trauma Surgery, Medical Faculty Mannheim of the University of Heidelberg, Mannheim, Germany; e Department of Traumatology and Department of Vascular Surgery, Oslo University Hospital, Oslo, Norway; f Department for Orthopaedics and Trauma Surgery, AUVA Trauma Center Salzburg, Salzburg, Austria; g Department of Trauma, Hand and Reconstructive Surgery, Saarland University Medical Center, Homburg, Germany.

**Keywords:** pelvic binder, pelvic fracture, survey, trauma surgery

## Abstract

Pelvic injuries mainly arise from high-energy traumas. The preclinical application of pelvic binders is advocated for suspected pelvic fractures with hemorrhage. In vitro cadaveric studies do not provide proof of principle despite their widespread use. This study evaluates clinical experience and physician perceptions regarding the use of pelvic binders in pelvic trauma. A total of 271 physicians regularly treating polytraumatized patients were surveyed using a 22-item multiple-choice questionnaire. Participants were recruited at trauma surgery conferences in 2019 and 2020 and via the mailing list of the German Society of Trauma Surgery. Data included respondents’ professional background and experience in the treatment of pelvic trauma. Most participants were experienced trauma surgeons, with 76.6% holding a consultant-level position and 68.6% having over 10 years of trauma experience. Pelvic binders were the most commonly used external stabilization device (90.4%), although only 48.9% believed they led to hemodynamic improvement in the majority of patients. Furthermore, 95% estimated that ≤ 50% of patients with pelvic fractures actually bled due to the fracture, and 88.5% reported that fewer than half of patients arriving with a binder had confirmed fractures. Despite this, 95.1% of respondents still felt pelvic binders were more effective than harmful. While pelvic binders remain widely accepted by clinicians, the survey findings indicate a disconnect between their routine use and the low observed rate of fracture-associated bleeding or confirmed pelvic injuries. These results suggest the need for more selective, evidence-based application of pelvic binders in the prehospital setting.

## 1. Introduction

### 1.1. Principle

The pelvis is a very stable bony scaffold; therefore, a relevant impact is needed to fracture it. Bleeding ensuing fracture of venous (85%) or arterial (15%) origin occurs almost exclusively after high-energy trauma in 10% to 30%,^[1]^ in cases where approximately one-third die from hemorrhagic shock.^[2]^ If bony pelvic- and hemodynamic instability are present without other sources of bleeding, current guidelines (Eastern Association for the Surgery of Trauma [EAST], Western Trauma Association, Advanced Trauma Life Support [ATLS], the World Society of Emergency Surgery, and Trauma Quality Improvement Project^[3]^) recommend the placement of pelvic binders. The rationale is that external compression may reduce fractures, increase intrapelvic pressure, and subsequently cause hemostasis.^[4]^ However, contradictory experimental and clinical findings challenge these recommendations.

### 1.2. Placement of indication for pelvic binders

First, in a study by Grant et al, testing for pelvic stability only yielded a specificity of 71% and sensitivity of 59 %.^[5]^ Second, hemodynamic instability can be misinterpreted in the environment of a crash site.^[6,7]^

### 1.3. No (experimental) proof of principle

In vitro research has shown that the intrapelvic space created by a pelvic ring fracture, even in massive diastasis, is much smaller than anticipated.^[8]^ The notion of reducing a pelvic fracture resulting in tamponade with subsequent hemostasis is based on the false assumption that a small pelvis is a closed anatomical space in which an open-book fracture is induced. External compression increased the intrapelvic pressure by <5 mm Hg after an equivalent of the entire human blood volume (5 liters) was experimentally infused.^[9]^ Therefore, if not self-limiting, bleeding following pelvic fracture needs to be stopped inside the hospital, either surgically by pelvic packing^[10]^ or interventionally by angioembolization.

### 1.4. Use of pelvic binders

In a study of 928 patients with suspected relevant injury to the pelvic region, only 7% actually incurred a pelvic fracture^[11]^ let alone a pelvic fracture causing bleeding. According to the above notion, only a minority of pelvic fractures (e.g., open-book fractures) potentially benefit from external compression, whereas it can worsen others (e.g., hip or acetabulum fractures). Ultimately, the application of pelvic binders was shown to be difficult in a study where more than half of the placements were performed incorrectly.^[12]^

Because of this conundrum, we conducted an online survey among peer clinicians to determine regional guidelines, available equipment, and experience with pelvic binders for suspected or proven pelvic injuries.

## 2. Methods

### 2.1. Study design and participants

This was a cross-sectional survey study designed to assess physician practices and attitudes toward pelvic binder use in pelvic trauma. Participants were recruited during 2 trauma surgery conferences hosted by the ATLS-Europe Association and the American College of Surgeons (Region 15) in Tallinn, Estonia (October 4–5, 2019, and October 16–17, 2020, the latter held online due to the COVID-19 pandemic). A QR code linking to the survey was displayed during presentations. In addition, an invitation email was sent via the mailing list of the German Society of Trauma Surgery on November 25, 2019 (valid email addresses: n = 9534).

### 2.2. Survey development

The questionnaire consisted of 22 multiple-choice questions administered via surveymonkey.com. The core survey was based on a previously published instrument by Wolfarth et al,^[13]^ which was adapted for an international audience. Additional items were included to capture institutional characteristics and individual physician experiences in managing pelvic trauma.

### 2.3. Data collection and analysis

All responses were anonymized and collected electronically. The study design did not permit inferential statistical analysis; therefore, results were summarized descriptively. Categorical variables were reported as absolute counts and percentages. The aggregated results are presented in Tables 1–4.

**Table 1:** Background of participants and their institutions.

		n								
1.	Country of workplace	271	Germany	Other	–	–	–	–	–	–
			85.2%	14.8%	–	–	–	–	–	–
2.	Trauma department no. of beds	271	<25	25–50	51–75	76–100	>100	–	–	–
			8.5%	30.6%	24.4%	17.3%	19.2%	–	–	–
3.	Trauma center status	271	Certified trauma center	No trauma center certification	–	–	–	–	–	–
			90%	10%	–	–	–	–	–	–
4.	Number of patients treated in trauma room per month in your department	271	none	1–5	6–10	11–15	>15	–	–	–
			1.1%	15.9%	17.3%	11.1%	54.6%	–	–	–
10.	Own position	269	Resident	Consultant	Attending	Head of department	Other	–	–	–
			19.3%	32%	13%	31.6%	4.1%	–	–	–
11.	Own experience in trauma care	270	<5 yr	5–10 yr	11–15 yr	16–20 yr	>20 yr	–	–	–
			10.4%	21.1%	22.2%	18.2%	28.2%	–	–	–
12.	Own certification status	270	ATLS® provider	ATLS® instructor	ATLS® director	PHTLS®	ITLS®	ETC®	equivalent	none
			60%	21.9%	8.9%	12.6%	2.2%	8.5%	10.4%	7.4%

ATLS® = advanced trauma life support, CT = computed tomography, ETC® = European Trauma Course, ICU = intensive care unit, ITLS® = International Trauma Life Support, PHTLS® = Pre Hospital Trauma Life Support, XR = plain X-ray.

**Table 2:** Available equipment and staff in the respective department.

		n					
5.	Does your hospital/department have a designated trauma team that treats severely injured patients in the trauma room?	271	Yes, always	Occasionally (depends on availability/ weekday/ time of day)	Never		
			81.9%	13.7%	4.4%	–	–
7.	Availability of angioembolisation	270	No	Yes, occasionally	Yes, 24/7	–	–
			21.1%	18.5%	60.4%	–	–
8.	Time to perform angioembolisation if available	270	Not available	<1 h	1–2 h	>2 h	–
			16.3%	48.9%	31.5%	3.3%	–
9.	Surgeons trained to perform pelvic packing in your department	270	0%	1%–25%	26%–50%	51%–75%	76%–100%
			5.9%	35.2%	24.8%	13.7%	20.4%

**Table 3:** Standard operating procedures.

		n					
6.	What medical specialty will indicate use of pelvic binder in your hospital/trauma room (multiple answers)?	270	Surgeon	Anesthesiologist	Emergency physician	Other	
			95.6%	24.4%	29.3%	9.3%	
13.	Trauma room: pelvic binder already put on patient preclinically -	270	We always evaluate stability again	We occasionally evaluate stability again	We never evaluate stability again		
			40.4%	36.3%	23.3%		
14.	Radiographic evaluation of suspected pelvic fracture	270	We use XR only	We use CT only	We use both		
			1.9%	33.7%	64.4%		
15.	In suspected pelvic fracture in order not to miss an open-book injury, we	270	Use XR with, then XR without pelvic binder	Use CT with, then XR without pelvic binder	Use CT with, then CT without pelvic binder	other	
			12.2%	48.9%	16.7%	22.2%	
16.	For initial treatment of pelvic instability + hemorrhagic shock without other source of bleeding, we use (multiple answers possible):	270	Pelvic binder	C-clamp	External fixator	Other	None
			90.4%	38.5%	65.2%	7.4%	0.7%
17.	Proven pelvic fracture with bleeding, binder on patient. When do you remove the binder?	268	After hemostasis	Shortly before osteosynthesis	No operation, no intervention, patient stable: on ICU <12 h	No operation, no intervention, patient stable: on ICU 12–24 h	No operation, no intervention, patient stable: on ICU >24 h
			17.9%	61.2%	5.2%	11.6%	4.1%

CT = computed tomography, ICU = intensive care unit, XR = plain X-ray.

**Table 4:** Experience with and outcomes using pelvic binders.

		n					
18.	Pelvic binder applied preclinically. What fraction of patients actually had a pelvic fracture proven by CT/XR?	262	0%	1%–25%	26%–50%	51%–75%	76%–100%
			1.9%	59.5%	27.1%	8.8%	2.7%
19.	Pelvic binder applied preclinically. What percentage of patients actually had bleeding due to pelvic fracture?	260	0%	1%–25%	26%–50%	51%–75%	76%–100%
			4.2%	80.8%	10%	4.2%	0.8%
20.	Pelvic binder applied preclinically. Proven pelvic fracture with pelvic bleeding. In what percentage did the pelvic binder improve circulation/haemostasis?	259	0%	1–25%	26–50%	51–75%	76–100%
			3.9%	27.8%	20.9%	32.1%	15.4%
21.	Pelvic binder applied preclinically. Proven pelvic fracture with pelvic bleeding. What percentage of patients did benefit from pelvic binder?	259	0%	1%–25%	26%–50%	51%–75%	76%–100%
			2.7%	20.1%	22.4%	29.7%	25.1%
22.	Do you feel that pelvic binders:	267	Do more harm than good	Do more good than harm			
			4.1%	95.9%			

CT = computed tomography, XR = plain X-ray.

### 2.4. Ethics approval

The study was approved by the Ethics Committee of the Medical Association of Rhineland Palatinate (approval no. 2019-14372) and was registered in the German Clinical Trials Register (registration no. DRKS00017683).

## 3. Results

### 3.1. Background of participants and their institutions (Table 1)

A total of 271 participants were recruited between November 2019 and November 2020. 85.2% worked in Germany, and more than half (60.9%) worked in a department with 50 or more beds for treating trauma patients. Almost all departments (90%) were certified trauma centers, and two-thirds (65.7%) treated more than 10 polytraumatized patients per month.

The vast majority (76.6%) of the participants were experienced physicians in the position of consultant by the very least, and 68.6% had more than 10 years of experience in the treatment of trauma patients. While 90.8% held an active ATLS certificate, PHTLS® (Pre Hospital Trauma Life Support) (12.6%), ITLS® (International Trauma Life Support) (2.2%), or ETC® (European Trauma Course) (8.5%) certificates were also common qualifications.

### 3.2. Available equipment and staff (Table 2)

In 81.9% of institutions, a designated trauma team was at hand for the treatment of polytraumatized patients. Angioembolization was available in 78.9%, either permanently (60.4%) or at least occasionally (18.5%) which took 80.4% <2 i.e. 48.9% <1 hour to perform. However, 65.9% answered that half or less of the surgeons in their department were trained to perform pelvic packing to stop venous bleeding. A multitude of external compression devices were used inside the hospital, of which pelvic binders (90.4%) were the most frequently used, but external fixators (65.2%) or C-clamps (38.5%) were employed.

### 3.3. Respective standard operating procedures (Table 3)

In most instances, a surgeon (95.4%), rather than an anesthesiologist (24.4%) or emergency physician (29.3%), indicated pelvic binder placement. In the case of an already applied binder, participants either occasionally (36.3%) or always (40.4%) tested for pelvic stability again. For radiographic evaluation of the respective pelvic injuries, X-ray evaluation was rarely used alone (1.9%), whereas the combination with computed tomography (CT) (64.4%) was the most frequently employed. In the case of a suspected open-book fracture, half (48.9%) used CT with, followed by X-ray without pelvic binder. In case of a proven pelvic fracture with bleeding, the binder was removed shortly before osteosynthesis in 61.2% of cases, rather than after hemostasis (17.9%). In the case of nonsurgical treatment, the patient was monitored in the intensive care unit; removal occurred before 12 hours in 5.2%, between 12 and 24 hours in 11.6%, and only after 24 hours in 4.1%.

### 3.4. Experience with pelvic binders (Table 4)

**Figure 1.**
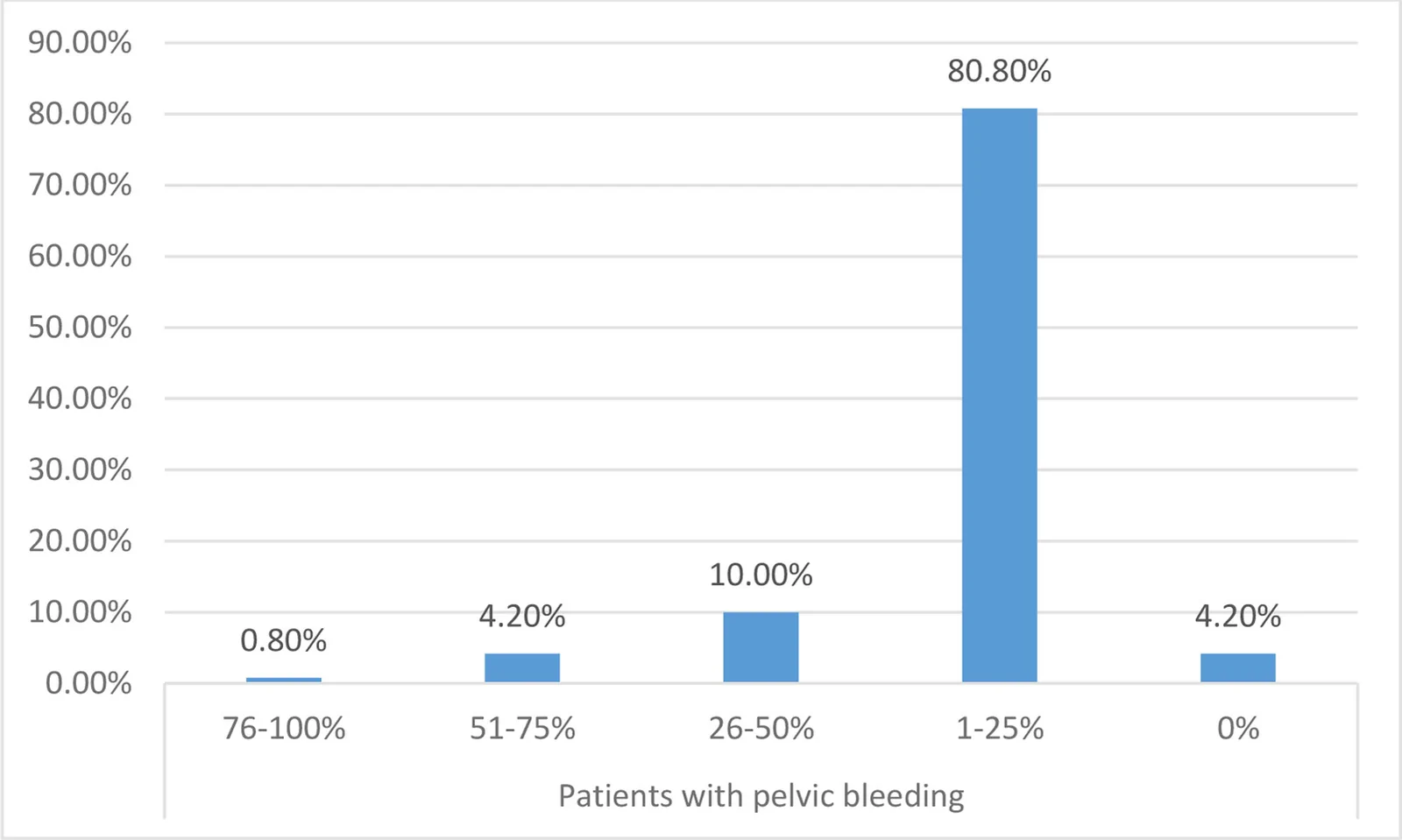
Bleeding following a pelvic fracture is encountered in less than a 4th of patients that were delivered to the trauma room wearing a pelvic binder.

**Figure 2.**
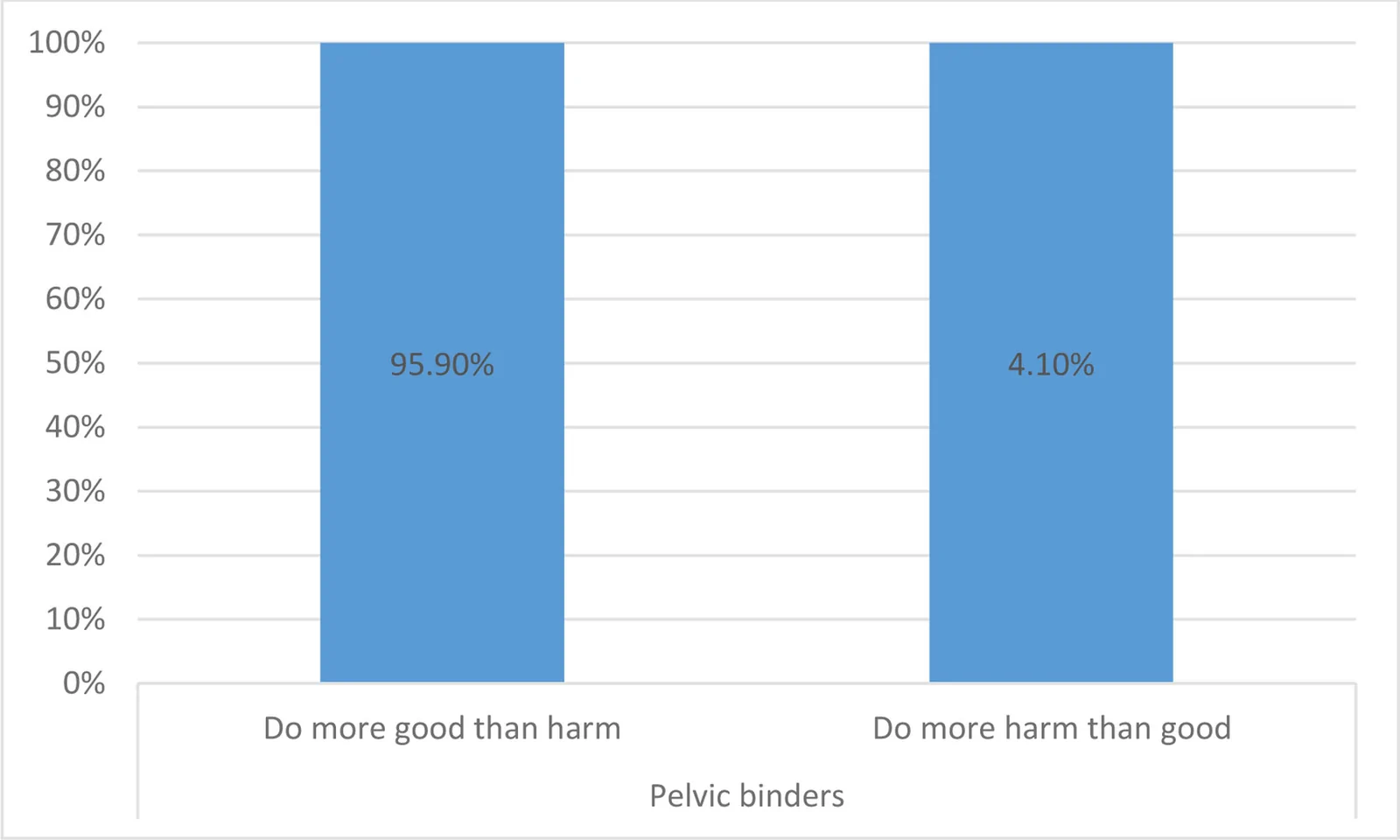
Although bleeding following pelvic fracture is a rare event, almost all physicians in our survey found the use of pelvic binders helpful.

In the case of a patient who delivered with a pelvic binder, 88.5% noted that less than half of the patients had actually incurred a pelvic fracture. In the case of a present pelvic fracture, 95% found that half or less (≤50%) of the patients bled due to the fracture (Fig. 1). In this case, 47.5% found that the majority (>50%) of patients improved hemodynamically through pelvic binding and 54.8% found that this majority benefited from pelvic binding. Ultimately, 95.1% found that pelvic binders were more effective than harmful (Fig. 2).

**Figure 3.**
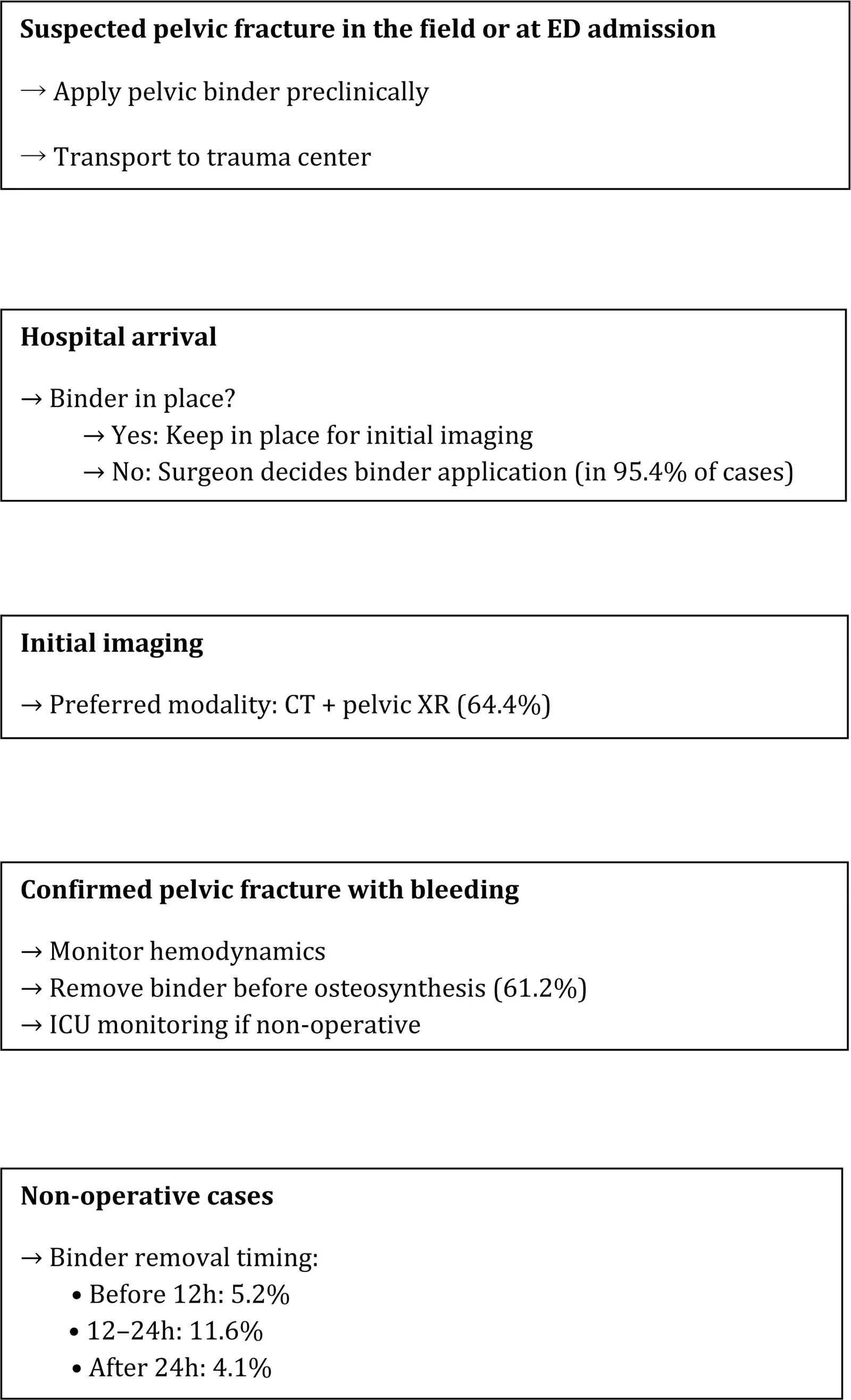
Proposed management protocol for suspected pelvic trauma (based on survey data). CT = computed tomography, ED = emergency department, ICU = intensive care unit, XR = plain X-ray.

Based on aggregated survey responses, a proposed management protocol reflecting real-world practice patterns is illustrated in Figure 3.

## 4. Discussion

### 4.1. Overuse

More than 80% of participating physicians reported that fewer than 1 in 4 patients actually exhibited bleeding due to pelvic injury, despite having received a pelvic binder. This suggests that the majority of patients were treated with external compression without clinical necessity. Experimental studies have shown that external compression fails to significantly increase intrapelvic pressure.^[8,9]^ Clinical studies on pelvic binders have produced conflicting results regarding their overall efficacy.^[14]^ Therefore, pelvic binders appear to be overused in current practice, a finding consistent with the observations of Trentzsch et al.^[15]^ Nevertheless, nearly all participants in our survey supported the continued use of binders. If external compression cannot meaningfully affect intrapelvic bleeding – as demonstrated in cadaveric models – then simple splinting with vacuum mattresses might suffice for emergency stabilization and safe transport of polytraumatized patients to the definitive care setting. Still, 95.9% of participants felt that their patients benefited from binder use, despite recent evidence suggesting that binders have no measurable effect on clinical outcomes, including survival.^[16,17]^

### 4.2. Placement of indication

Once a patient is delivered to the trauma room with a binder already in place, most participants reported retesting pelvic stability, despite the well-documented low sensitivity and specificity of this maneuver.^[5,18]^ In our study, 76.7% of respondents stated they always or occasionally retested for stability – a practice also observed by Wohlrath et al,^[13]^ who reported a similar rate of 91%. However, given that imaging (e.g., FAST or whole-body CT) is the immediate next step in trauma care, such manual testing may be redundant, especially once a binder has already been applied.

### 4.3. Treatment capabilities

Our findings indicate that most institutions represented in this survey possess adequate personnel and experience to manage polytraumatized patients. However, 21.1% of participants reported lacking permanent access to arterial hemorrhage control capabilities, and another 18.5% lacked consistent access to angioembolization services. More concerning, approximately 2-thirds of institutions reported that their surgeons were not trained in pelvic packing – the key intervention for managing venous bleeding, which accounts for the majority (~85%) of pelvic hemorrhages.^[10]^ Consequently, a significant proportion of institutions may be underprepared to manage the most common bleeding sources in pelvic trauma.

### 4.4. Clinical implications

Our findings highlight a considerable mismatch between perceived utility and actual clinical effectiveness of pelvic binders. Although most participants believed their patients benefitted from binder application, the reported incidence of bleeding in patients with pelvic fractures was relatively low, and existing experimental studies question the mechanical rationale for compression-based hemorrhage control. This discrepancy suggests a potential overreliance on pelvic binders, likely influenced by tradition, perceived safety, and guideline momentum rather than robust outcome data. Given that most surveyed trauma centers possess sufficient diagnostic capabilities and resources, reevaluating the role of external compression – especially when venous bleeding cannot be managed surgically in a timely manner – may be warranted.

While this study was not intended to establish clinical guidelines, the reported practice patterns allowed us to outline a simplified management protocol (Fig. 3). This diagram is meant to summarize prevalent real-world approaches to pelvic binder use as described by experienced trauma surgeons across Europe. It may serve as a foundation for further prospective evaluations or guideline development.

### 4.5. Limitations and strengths

This study has several limitations inherent to its design. First, it relies on self-reported data from physicians, which may introduce recall bias and social desirability bias, potentially overestimating adherence to best practices or underreporting of complications. The absence of objective clinical data and the descriptive nature of the analysis prevent conclusions about clinical efficacy or causality. Additionally, most participants were based in German-speaking regions, which may limit the broader international applicability of the findings. The survey instrument was adapted from a previously published national version, which, while useful for standardization, may not capture all nuances of international practices.

Despite these limitations, the study also has notable strengths. It includes a large and highly experienced cohort of trauma care providers, most of whom work in certified trauma centers with high case volumes. This enhances the practical relevance and ecological validity of the findings. Furthermore, the study is one of the few to systematically capture clinical perspectives on pelvic binder use across diverse institutions, providing valuable insight into real-world treatment patterns and potential gaps in care. Finally, it highlights both the perceived value and potential overuse of pelvic binders, offering a foundation for more targeted future studies.

## 5. Conclusions

This study challenges current pelvic trauma practices by showing that bleeding is rare and clinical benefit of binders remains unproven. Despite widespread use, evidence for efficacy is lacking, and many applications may be unnecessary. A reassessment of their role is warranted, especially in systems lacking definitive hemorrhage control options.

## Author contributions

**Conceptualization:** Roman Klein.

**Data curation:** Roman Klein.

**Formal analysis:** Roman Klein.

**Methodology:** Roman Klein.

**Software:** Roman Klein.

**Validation:** Roman Klein.

**Writing – original draft:** Roman Klein, Nikolai Ramadanov.

**Writing – review & editing:** Nikolai Ramadanov, Ali Darwich, Joakim Jørgensen, Arnold J. Suda, Tim Pohlemann, Christoph Wölfl.
